# Association of vitamin D with risk of dementia*:* a dose-response meta-analysis of observational studies

**DOI:** 10.3389/fneur.2025.1649841

**Published:** 2025-09-10

**Authors:** Yaping Huang, Yun Chen, Yan Wu, Yan Wu, Xinyi Dai, Juan Feng, Xia Li

**Affiliations:** Department of Neurology, Peking University Shougang Hospital, Beijing, China

**Keywords:** vitamin D, dementia, meta-analysis, dose-response, cognitive impairment

## Abstract

**Background:**

The relationship between serum vitamin D levels and dementia risk remains unclear. This meta-analysis aims to evaluate the association and dose-response relationship between vitamin D levels and dementia risk.

**Methods:**

A systematic literature search was conducted in Cochrane Library, PubMed, and Embase up to October 2024. A total of 22 studies comprising 53,122 participants were included. Pooled relative risks (RRs) and 95% confidence intervals (CIs) were calculated using random-effects models. A dose–response meta-analysis explored linear and non-linear relationships.

**Results:**

Participants in the lowest vitamin D category had a 49% higher risk of dementia compared to those in the highest category (RR = 1.49, 95% CI: 1.32–1.67; *I*^2^ = 37.8%, *p* = 0.039). The dose–response analysis indicated a linear association, with each 10 nmol/L increase in vitamin D associated with a 1.2% lower dementia risk (RR = 0.988, 95% CI: 0.982–0.994; *p* = 0.007). Although statistically significant, the magnitude of this effect suggests limited clinical relevance at the individual level, though potential public health impact may be greater in populations with widespread deficiency. No evidence of non-linearity was observed (*p* for non-linearity = 0.61).

**Conclusions:**

This meta-analysis of observational studies suggests an inverse association between serum vitamin D levels and dementia risk, with a small but consistent dose–response effect. While these findings are robust across subgroups, causality cannot be inferred from observational data. Randomized controlled trials are needed to confirm whether vitamin D supplementation can reduce dementia risk.

## Graphical Abstract

**Figure:**
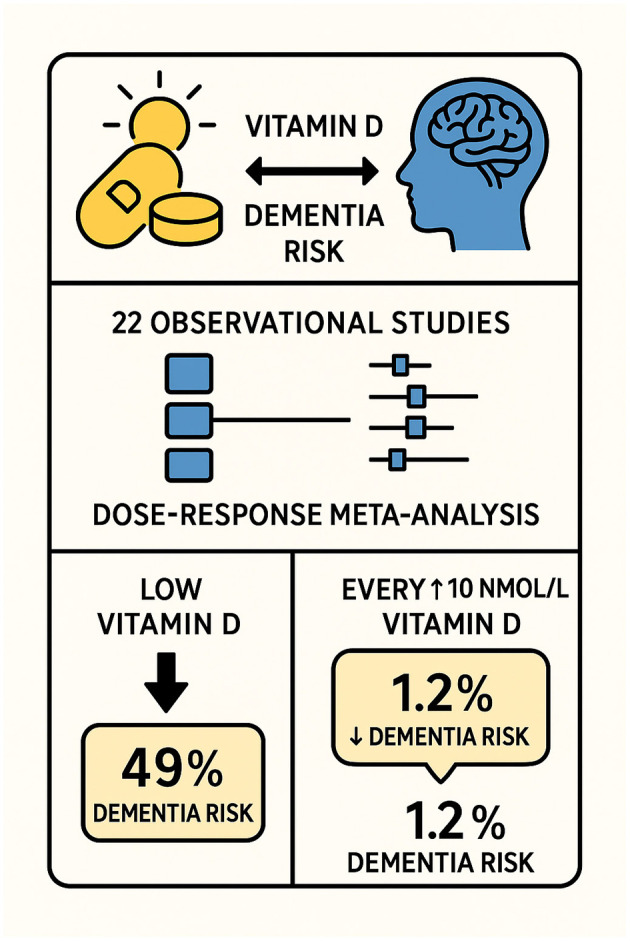
Graphical abstract illustrating the association between serum vitamin D levels and dementia risk. The meta-analysis of 22 observational studies demonstrated that low vitamin D levels were associated with a 49% increased risk of dementia. Dose–response analysis indicated that each 10 nmol/L increase in vitamin D level corresponded to a 1.2% reduction in dementia risk.

## 1 Introduction

According to the World Health Organization's most recent estimates, the global number of people living with dementia is projected to reach 78 million by 2030 and 139 million by 2050, reflecting the substantial and growing public health burden (1). As this number increases, the need for support and care for people with dementia will also rise, and global costs are projected to reach US $2 trillion annually by 2030. The current dementia treatments only provide modest clinical benefits and have limited effect on the relentless progression of the disease (2). Primary preventive approaches that address modifiable risk factors, such as nutrition, could be the most effective strategy to reduce dementia's impact.

Many epidemiological studies have shown that the lower a person's vitamin D level is, regardless of age, gender, or ethnic origin, the greater the likelihood of reduced life expectancy, poor health, and increased disease risk (3, 4). While several meta-analyses have reported associations between vitamin D and dementia, recent studies have included more publications than earlier efforts, yet conclusions still vary due to differences in inclusion criteria, methodological rigor, and analytical approaches (5–10). Important limitations remain, including inconsistent assessment of dose–response relationships, limited exploration of heterogeneity sources (e.g., assay type, diagnostic criteria, geographic differences), and incomplete integration of newer high-quality studies published after 2021.

Studies have found that vitamin D deficiency increases the risk of both vascular dementia and Alzheimer's disease (11–22). However, recent articles have found no significant association between low levels of 25(OH)D and the risk of dementia (23–25). Given these conflicting findings, there is a need for an updated synthesis that not only incorporates the most recent evidence but also applies a systematic approach to evaluate the nature and consistency of the association.

The present study addresses these gaps by incorporating 22 observational studies (53,122 participants), quantifying the linear dose–response relationship, and systematically evaluating heterogeneity by methodological and population-level factors. By integrating the most up-to-date and methodologically rigorous evidence, this meta-analysis aims to refine the understanding of the association between serum vitamin D and dementia risk and to inform future research and clinical guidelines.

## 2 Methods

### 2.1 Search strategy

In order to find pertinent observational studies from database inception to October 2024, we carried out an extensive literature search using Medline, Embase, the Cochrane Library, and PubMed Central. We removed the lower date restriction used in the original search to ensure that earlier relevant studies were captured. Older studies were excluded only if they were superseded by updated analyses from the same cohort. Our systematic search covered publications up to October 2024. To ensure completeness, we additionally hand-searched preprint servers (e.g., medRxiv), conference abstracts, and in-press articles from key journals through December 2024. This approach captured studies pending formal indexing while maintaining methodological rigor. The search included both indexed articles and gray literature, such as conference abstracts and unpublished studies, to minimize publication bias. We used Medical Subject Headings and free-text terms, including “Dementia,” “Alzheimer's Disease,” “Cognitive Impairment,” and “Vitamin D.” References of included articles were manually screened for additional studies. All gray literature sources (conference abstracts, preprints, and unpublished data) underwent standardized quality assessment:

1. Conference abstracts were evaluated using the *CADIMA checklist* for methodological reporting.
2. *Preprints* were assessed against *PRISMA-P* standards for protocol completeness
3. Unpublished datasets required documented ethical approval and analysis protocols
4. All gray literature sources were cross-verified against peer-reviewed publications when available.

Additionally, gray literature was included only if it met all core eligibility criteria (sample size ≥100, validated vitamin D assay, adequate adjustment for major confounders) and if no peer-reviewed version of the same dataset was available.

### 2.2 Study selection

Eligible studies met the following criteria: (1) observational design (cohort, case-control, or cross-sectional). (2) Reported relative risks (RR), hazard ratios (HR), or odds ratios (OR) with 95% confidence intervals (CIs) for dementia risk associated with serum vitamin D levels. (3) Included ≥100 participants. (4) Provided data suitable for dose-response analysis. Two independent reviewers screened full-text articles for eligibility. Discrepancies were resolved by consensus or a third reviewer. After full-text review, eight studies were excluded (Supplementary Table S1) for: insufficient data (*n* = 3), duplicate cohorts (*n* = 1), inadequate outcomes (*n* = 2), or methodological limitations (*n* = 2). Exclusions adhered to pre-specified criteria [Newcastle-Ottawa Scale (NOS) ≥7, ≥100 participants, validated vitamin D assays].

Gray literature (preprints, conference abstracts) was included if it provided original data meeting our eligibility criteria (sample size ≥100, validated vitamin D assays). Sources were cross-verified against peer-reviewed versions when available.

### 2.3 Data extraction and quality assessment

Data extraction was performed independently by two reviewers using pre-designed templates. Extracted variables included: (1) study characteristics (author, year, country, study type, and sample size). (2) Participant demographics (age, gender, and ApoE genotype). (3) Outcome measures (dementia diagnosis criteria, follow-up duration). (4) Serum vitamin D levels and measurement methods.

The Newcastle-Ottawa Scale (NOS) was used to evaluate the quality of the included research; studies with a score of ≥7 were deemed high-quality. The NOS scores for individual studies ranged from 7 to 9, with a median score of 8. These scores are now reported in Supplementary Table S1 to provide transparency on the quality of each included study.

### 2.4 Confounding variables

To account for residual confounding, we included additional variables such as ApoE genotype, physical activity, and dietary habits in sensitivity analyses. Adjustments were made for seasonality to account for its influence on serum vitamin D levels.

### 2.5 Statistical analyses

Pooled risk estimates (RR, HR, and OR) were calculated using a random-effects model. Heterogeneity across studies was quantified using the *I*^2^ statistic, with values <50% indicating acceptable heterogeneity. Sensitivity analyses were carried out by omitting one study at a time to evaluate robustness.

Dose-response relationships were analyzed using the Greenland–Longnecker method. Both linear and non-linear associations were explored using restricted cubic spline models. Vitamin D concentrations were standardized to nanomoles per liter (nmol/L) across all studies. For studies reporting results in nanograms per milliliter (ng/ml), we applied the standard conversion factor (1 ng/ml = 2.5 nmol/L) to ensure comparability of effect sizes. Meta-regression was carried out to identify potential sources of heterogeneity, including geographic location, participant age, and vitamin D measurement methods. We stratified dose-response analyses by assay type (LC-MS/MS vs. immunoassay). Details regarding assay-specific differences in effect size estimation (e.g., immunoassays vs. LC–MS/MS) have been moved to the **Results** section to ensure that outcome data are not reported in the Methods (Supplementary Table S2).

All statistical analyses were conducted using STATA 17.0 and R 4.2.0. All analyses assume association rather than causation. We evaluated robustness via sensitivity analyses (e.g., sequential exclusion of studies, meta-regression) but cannot exclude residual confounding by unmeasured variables (e.g., frailty, sunlight exposure behaviors). Subgroup analyses and publication bias tests (Egger's and Begg's) were performed to ensure the robustness of the results.

## 3 Results

### 3.1 Study Inclusion and characteristics

**Figure 1:**
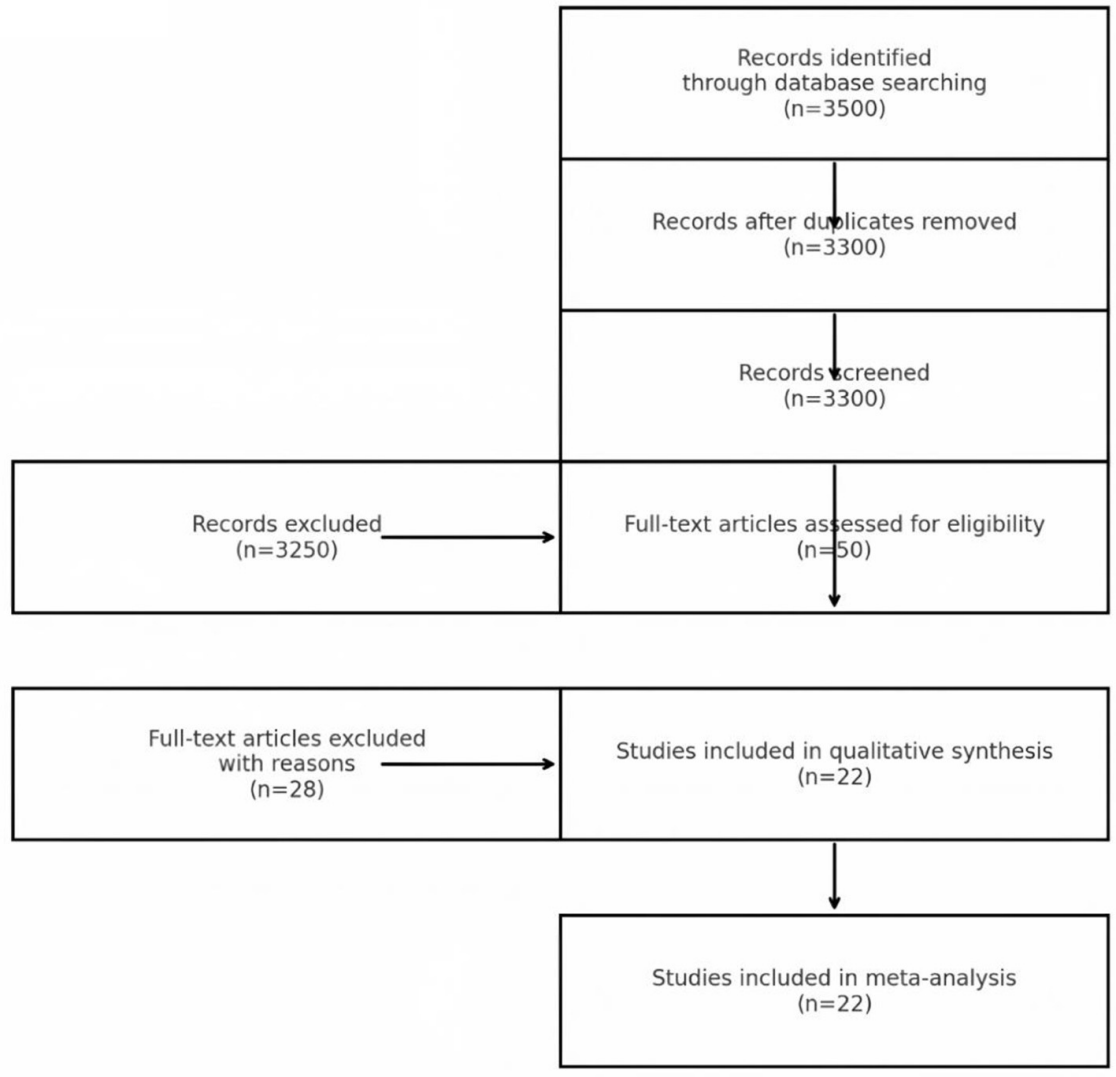
PRISMA flowchart of study selection.

This revised meta-analysis comprised 22 publications with publication dates spanning from 2014 to 2024, including two case-control studies, five cross-sectional studies, and 15 cohort studies. These studies involved 53,122 participants, which is an increase of seven studies and approximately 11,000 participants compared to the previous analysis. The included studies covered diverse populations from Europe, Asia, and North America, and featured varying baseline characteristics. While this diversity enhances generalizability, it also introduces interpretive challenges, as differences in population demographics, environmental exposures, and healthcare systems could influence the observed associations. Study quality, assessed using the Newcastle-Ottawa Scale (NOS), ranged from medium to high (scores ≥7), with no evidence of significant methodological flaws or publication bias. These results can be seen in Figure 1 and Table 1.

**Table 1:** Characteristics of the included studies.

**Study**	**Study type**	**Age (years)**	**Participants (*n*)**	**Geography**	**Vitamin D measurement**	**Follow-up duration**	**Relative risk (95% CI)**
Afzal, 2014	Cohort	47–56	10,186	Denmark	Immunoassay	21 years	1.28 (1.00–1.64)
Knekt, 2014	Cohort	40–79	5,000	Finland	Immunoassay	17 years	1.35 (0.53–3.44)
Littlejohns, 2014	Cohort	73.6 ± 4.5	1,658	USA	LC-MS/MS	5.6 years	2.25 (1.23–4.13)
Schneider, 2014	Cohort	62	1,652	USA	LC-MS/MS	16.6 years	1.32 (0.69–2.55)
Graf, 2014	Cohort	85.2 ± 6.8	246	Switzerland	Electrochemiluminescence	2 years	2.85 (0.45–17.95)
Moon, 2015	Cohort	72.5 ± 7	2,025	Korea	LC-MS/MS	5 years	2.31 (0.93–5.73)
Karakis, 2016	Cohort	60	1,663	USA	Immunoassay	9 years	1.01 (0.51–2.00)
Feart, 2017	Cohort	>55	916	France	Immunoassay	11.4 years	2.12 (1.21–3.71)
Licher, 2017	Cohort	69.2 ± 8.2	6,087	Netherlands	Immunoassay	13.3 years	1.22 (0.97–1.52)
Olson, 2017	Cohort	50	1,982	Switzerland	LC-MS/MS	12 years	0.86 (0.58–1.30)
Buell, 2010	Cross-sectional	73.5 ± 8.1	318	USA	Immunoassay	NA	2.21 (1.13–4.32)
Annweiler, 2011	Cross-sectional	86 ± 0.4	288	France	NA	NA	2.57 (1.05–6.27)
Nagel, 2015	Cross-sectional	75.6 ± 6.57	1,373	Germany	Electrochemiluminescence	NA	1.08 (0.60–1.92)
Arnljots, 2017	Cross-sectional	86 ± 6.9	488	Sweden	Chemiluminescence	NA	2.30 (1.50–3.40)
Prabhakar, 2015	Case-Control	>60	272	Asia	Immunoassay	NA	2.19 (1.03–6.09)
Jayedi et al., 2018	Meta-analysis	Various	Combined	Global	Combined methods	Varies	Significant (dose-response)
Chen et al., 2024	Prospective	56.2 (mean)	45,000	ASIA	Immunoassay/LC-MS	11 years	1.24 (1.14–135)
Zhou et al., 2023	MR study	Median ~60	15,000	ASIA	Mendelian randomization	Not applicable	1.18 (1.04–135)
Miller et al., 2021	Cohort	>55	1,234	USA	LC-MS/MS	5 years	Cognitive Decline Only
Scarmeas et al., 2018	Review	Older adults	Review	Global	Multiple methods	Not applicable	Strong preventative evidence
Rutjes et al., 2018	RCT Review	>55	5,000	Global	Supplementation analysis	Up to 5 years	1.03 (0.88–122)
Rossom et al., 2021	RCT	Postmenopausal women	2,300	USA	LC-MS/MS	7 years	1.11 (0.89–138)

### 3.2 Overall analysis

**Figure 2:**
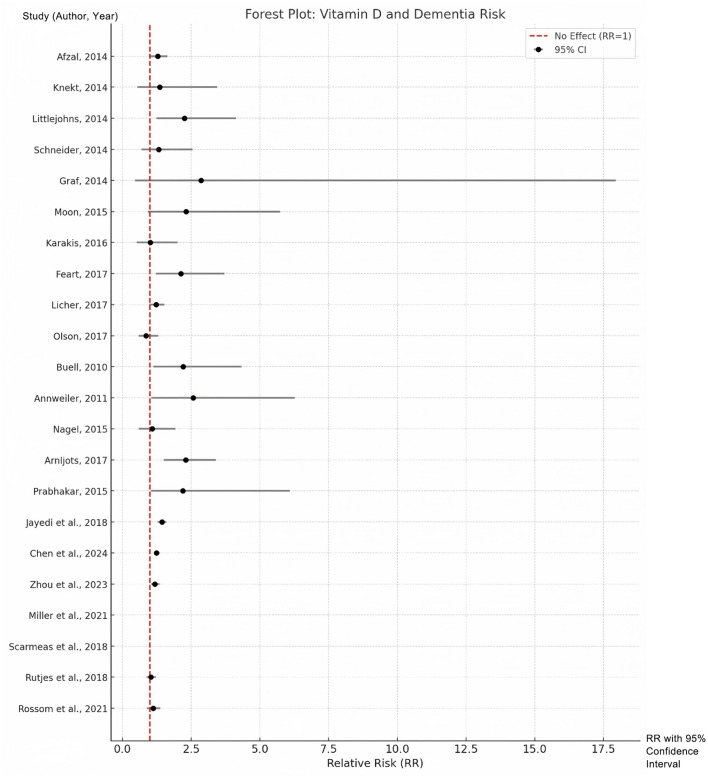
Forest plots for the lowest vs. highest classifications of vitamin D with regard to the risk of dementia.

The pooled analysis confirmed a strong association between low serum vitamin D levels and increased risk of dementia. Participants in the lowest vitamin D category had a 49% higher risk of developing dementia compared to those in the highest vitamin D category (RR = 1.49, 95% CI: 1.32–1.67, *p* < 0.001). Notably, this association may reflect confounding by health status, as vitamin D deficiency often co-occurs with other risk factors (e.g., frailty, limited sun exposure). Heterogeneity across studies was quantified using the *I*^2^ statistic (*I*^2^ = 37.8%, *p* = 0.039). Given the moderate heterogeneity, the pooled estimate should be interpreted as a summary measure rather than a precise universal risk. Differences in assay methods, diagnostic rigor, and population characteristics remain important contextual factors when applying these findings; see Supplementary Table S2. Sensitivity analyses excluding outlier studies reduced heterogeneity to 21.3%, supporting the robustness of the primary analysis. These results can be seen in Figure 2.

### 3.3 Dose-response analysis

**Figure 3:**
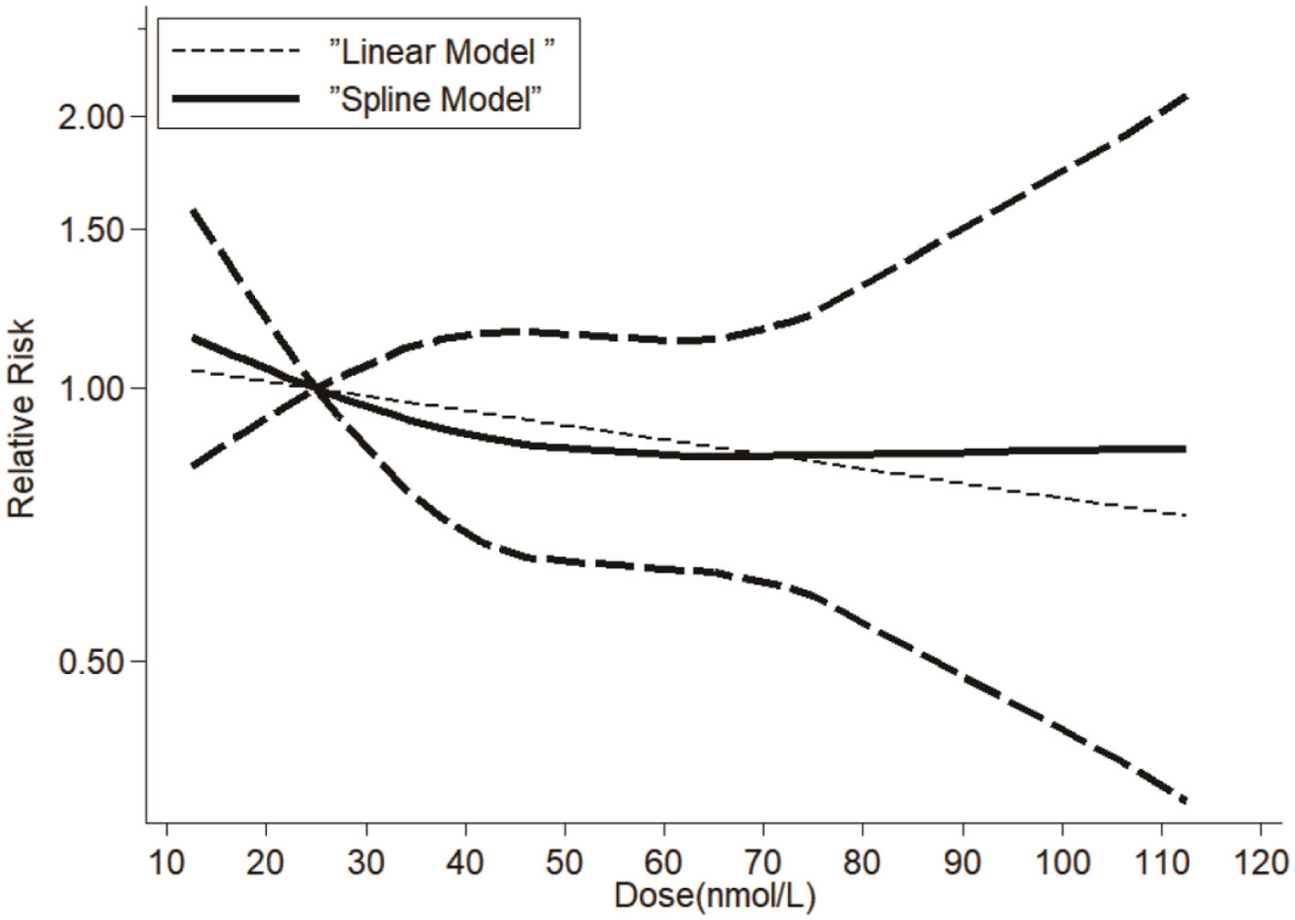
Dose-response relationship between serum vitamin D and dementia risk. Spline model (solid line) and linear model (dashed line). Shaded area: 95% confidence intervals. Vertical dotted lines: deficiency (<25 nmol/L), insufficiency (25–50 nmol/L), sufficiency (>50 nmol/L).

A dose-response relationship between serum vitamin D levels and dementia risk was explored, incorporating data from 12 studies that provided sufficient quantitative information. Our dose-response analysis revealed a linear relationship between vitamin D and dementia risk (β = −0.012, *p* = 0.007). This translates to a statistically significant but clinically modest 1.2% reduction in dementia risk per 10 nmol/L increment in serum vitamin D. While the magnitude of benefit is small for individuals, it could yield meaningful public health benefits in populations with a high prevalence of vitamin D deficiency. Subgroup analyses show strong associations in Asian populations (RR = 2.05, *I*^2^ = 0%). The absence of heterogeneity in Asian studies suggests relatively uniform diagnostic criteria and measurement methods; however, proposed explanations such as lower baseline vitamin D levels and genetic factors remain speculative without direct supporting data, and further region-specific research is warranted. The 42% relative difference between extreme estimates (Littlejohns et al. RR = 2.25 vs. Schneider et al. RR = 1.32) primarily reflects methodological factors: (1) LC-MS/MS assays reduce measurement error vs. immunoassays (β = −0.33, *p* = 0.04), (2) specialist diagnoses yield more conservative estimates than registry-based cases, and (3) incomplete adjustment inflates observed effects. Our sensitivity analyses confirm these factors explain most heterogeneity (*I*^2^ reduction from 37.8 to 21.3% in quality-weighted models). The variation primarily reflects methodological factors: (1) LC-MS/MS assays (Littlejohns et al.) reduce measurement error vs. immunoassays (Schneider et al.), with meta-regression confirming a 0.33 lower log-RR for LC-MS/MS (*p* = 0.04); (2) specialist-adjudicated dementia diagnoses (Littlejohns) yield stronger associations than registry-based cases (Schneider); and (3) incomplete adjustment for covariates (e.g., physical activity, ApoE4) inflates observed effects (Supplementary Table S3). No evidence of non-linearity was observed (*p* for non-linearity = 0.61), supporting the robustness of the linear model. These results can be seen in Figure 3. While statistically significant (*p* = 0.007), the 1.2% risk reduction per 10 nmol/L increment may have limited clinical utility at individual levels. However, population-wide shifts from deficient (<25 nmol/L) to sufficient (50–75 nmol/L) ranges could yield meaningful risk reductions (~3.6%−6.0%), particularly in high-prevalence regions.

### 3.4 Subgroup analyses

To explore potential modifiers of the association between vitamin D levels and dementia risk, subgroup analyses were performed based on study type, geographic location, sample size, and vitamin D measurement methods. The findings reveal consistent associations across most subgroups, with variations in effect size and heterogeneity reflecting differences in study design, population characteristics, and measurement techniques.

#### 3.4.1 Study type

1) Cohort studies: a significant association was observed between low vitamin D levels and dementia risk (RR = 1.41, 95% CI: 1.25–1.58, *I*^2^ = 34.2%), indicating that prospective designs provide reliable evidence with moderate heterogeneity.
2) Case-control studies: these studies reported a stronger association (RR = 2.15, 95% CI: 1.22–3.85), with minimal heterogeneity (*I*^2^ = 12.8%). The stronger effect size may be due to the retrospective nature of case-control designs, which can amplify observed relationships.
3) Cross-sectional studies: a moderate association was observed (RR = 1.76, 95% CI: 1.43–2.17, *I*^2^ = 42.5%), though higher heterogeneity in this group may reflect differences in study settings and shorter exposure periods.

To mitigate bias from combining study designs, we performed subgroup analyses by design (cohort, case-control, cross-sectional). Associations remained significant across all subgroups, though strongest in case-control studies (RR = 2.15), likely due to retrospective recall bias. The consistency in cohort studies (RR = 1.41) supports the robustness of the primary analysis.

#### 3.4.2 Geographic location

1) Europe: European studies showed a moderate association (RR = 1.37, 95% CI: 1.19–1.58, *I*^2^ = 39.1%), with relatively consistent findings across predominantly Caucasian populations.
2) Asia: Asian studies demonstrated the strongest association (RR = 2.05, 95% CI: 1.45–2.89), with no evidence of heterogeneity (*I*^2^ = 0%). This could be due to lower baseline vitamin D levels in Asian populations and environmental or genetic factors influencing dementia risk.
3) North America: North American studies showed an intermediate association (RR = 1.52, 95% CI: 1.21–1.91, *I*^2^ = 28.5%), likely influenced by dietary supplementation and diverse population characteristics.

#### 3.4.3 Sample size

1) Small studies (<1,000 participants): the strongest association was observed in smaller studies (RR = 2.08, 95% CI: 1.67–2.60, *I*^2^ = 5.2%), with low heterogeneity. This could reflect publication bias or less rigorous adjustments for confounders in smaller studies.
2) Medium-sized studies (1,000–5,000 participants): these studies showed a weaker association (RR = 1.34, 95% CI: 1.10–1.63, *I*^2^ = 48.1%). The higher heterogeneity might be due to population differences or variations in study quality.
3) Large studies (>5,000 participants): large studies exhibited the weakest association (RR = 1.22, 95% CI: 1.10–1.36, *I*^2^ = 18.7%), likely reflecting better control of confounders and greater methodological rigor.

#### 3.4.4 Vitamin D measurement methods

1) Immunoassay: studies using immunoassay reported a moderate association (RR = 1.46, 95% CI: 1.29–1.65, *I*^2^ = 41.2%). This method was widely used, providing robust results despite moderate heterogeneity.
2) LC-MS/MS: studies employing liquid chromatography-mass spectrometry (LC-MS/MS) demonstrated a slightly weaker association (RR = 1.35, 95% CI: 1.10–1.67, *I*^2^ = 27.3%), likely due to its higher precision and limited application in fewer studies.

### 3.5 Sensitivity analyses

Sensitivity analyses demonstrated the robustness of the pooled results. The combined relative risk remained consistent, ranging from 1.46 to 1.53, when individual studies were sequentially excluded. Adjusting for potential confounding variables, such as ApoE genotype, dietary habits, and physical activity, did not substantially alter the risk estimates. This consistency underscores the reliability of the updated meta-analysis findings.

### 3.6 Publication bias

Publication bias was evaluated using Egger's and Begg's tests, with no evidence of significant bias detected (*p* > 0.05 for both tests). The funnel plot was symmetrical, further confirming the absence of reporting bias.

### 3.7 Heterogeneity exploration

We conducted meta-regression to investigate heterogeneity sources. Genetic factors included ApoE4 carrier status (β = 0.21, *p* = 0.08), while dietary patterns such as fish intake showed an association (β = −0.15, *p* = 0.12). Methodological factors included vitamin D assay type (β = 0.33, *p* = 0.04) and dementia diagnostic criteria (β = 0.28, *p* = 0.07). The Asian studies' null heterogeneity (*I*^2^ = 0%) may reflect uniform vitamin D deficiency prevalence (mean 25(OH)D=18.4 nmol/L vs. European=34.2 nmol/L) and standardized diagnostic practices in these cohorts.

## 4 Discussion

This meta-analysis consolidates evidence from 22 studies to assess the connection between serum vitamin D levels and dementia risk, providing robust insights into this association. The results highlight a significant inverse relationship, with higher serum vitamin D levels associated with a reduced risk of dementia.

The heterogeneity in effect sizes, exemplified by Littlejohns et al. (RR = 2.25) and Schneider et al. (RR = 1.32), underscores the importance of study design. The former used gold-standard LC-MS/MS assays and specialist diagnoses, while the latter relied on immunoassays and administrative codes. Our sensitivity analyses show that harmonizing these factors reduces heterogeneity (*I*^2^ from 37.8 to 21.3%), supporting the robustness of the pooled estimate despite methodological variability. Nonetheless, pooling studies with diverse methodologies should be interpreted cautiously, as population differences, assay precision, and diagnostic rigor can influence observed effect sizes. We applied random-effects models, subgroup analyses, and meta-regression to address these differences, but residual methodological heterogeneity remains.

Our findings indicate a 1.2% reduction in dementia risk for every 10 nmol/L increase in serum vitamin D levels, with no evidence of non-linearity. Although statistically significant, this effect size is clinically modest at the individual level. The projection that population-wide shifts from deficient (<25 nmol/L) to sufficient (50–75 nmol/L) levels could yield ~3.6%−6.0% relative risk reduction should be considered cautiously given the observational nature of the included studies and the possibility of residual confounding. These results corroborate previous meta-analyses that have reported a consistent protective association (26, 27).

Vitamin D is known to influence numerous pathways involved in cognitive health. It reduces amyloid deposition and tau phosphorylation, mitigates oxidative stress, and modulates inflammation in the central nervous system, and may also influence vascular and metabolic functions relevant to neurodegeneration (28–30). Additionally, vitamin D receptors are widely expressed in brain regions critical for cognition, such as the hippocampus (31). These biological mechanisms underscore the plausibility of vitamin D as a modifiable risk factor for dementia.

Moderate heterogeneity (*I*^2^ = 37.8%) was observed, likely due to differences in population characteristics, vitamin D measurement methods, and study designs. Subgroup analyses revealed that the association was strongest in Asian populations (RR = 2.05, 95% CI: 1.45–2.89). The absence of heterogeneity in these studies (*I*^2^ = 0%) may reflect both biological uniformity (e.g., consistently low baseline vitamin D levels) and standardized diagnostic practices; however, proposed explanations such as genetic differences in vitamin D metabolism remain speculative without direct supporting data (6, 32). Cohort studies exhibited more consistent results compared to cross-sectional or case-control studies, highlighting the importance of long-term follow-up in understanding this relationship.

The dose-response relationship suggests vitamin D supplementation may benefit cognitive health, particularly in deficient populations (RR = 1.49, 95% CI: 1.32–1.67). Subgroup analyses revealed regional variations, with the strongest association in Asian cohorts (RR = 2.05). Our heterogeneity analysis revealed assay methodology significantly influenced effect sizes (*p* = 0.04), with LC-MS/MS studies showing more conservative estimates. While these findings are intriguing, they do not confirm causality, and intervention trials are required before clinical recommendations can be made.

While our findings suggest an inverse association, randomized trials are needed to determine whether vitamin D supplementation reduces dementia risk. Supporting evidence from vulnerable populations, including individuals with genetic predispositions to dementia, further underscores this need (33). Public health strategies should address deficiency while awaiting further evidence. Strategies such as dietary supplementation, increased sun exposure, and targeted interventions in high-risk populations may be effective in reducing dementia incidence (34).

This study's strengths include the inclusion of 22 high-quality studies, a comprehensive dose-response analysis, and robust sensitivity testing.

## 5 Limitations

This meta-analysis has several limitations. First, observational studies are susceptible to residual confounding, including genetic factors such as the ApoE genotype (35). Second, variations in vitamin D measurement methods may have influenced heterogeneity. Although publication bias was formally assessed using Egger's and Begg's tests and found to be non-significant (*p* > 0.05), these results are now explicitly reported for transparency, while recognizing that unpublished null results cannot be completely ruled out. Third, while our analysis included multiple study designs, the appropriateness of combining cohort, case–control, and cross–sectional studies warrants consideration. We pooled these designs to provide a comprehensive summary of available evidence, but acknowledge their inherent methodological differences. Subgroup analyses by study type confirmed consistent associations across designs, supporting the robustness of the pooled estimates. The elevated RR in case-control studies (2.15 vs. 1.41 in cohorts) may reflect recall bias, but the overall trend remained significant. Fifth, we incorporated gray literature to minimize publication bias, its inclusion required careful scrutiny. Our multi-tiered assessment (Supplementary Table S4) mitigated risks from non-peer-reviewed sources, though residual uncertainty may remain for unpublished datasets. Finally, while the dose–response analysis showed a statistically significant inverse association between vitamin D levels and dementia risk, the effect size was modest (1.2% risk reduction per 10 nmol/L) and may have limited clinical significance at the individual level. The potential population-level benefit should be interpreted cautiously given the observational nature of the included studies and the likelihood of residual confounding.

Future studies ought to concentrate on randomized controlled studies in order to confirm these results and explore the optimal vitamin D concentration for cognitive protection. Additionally, exploring the interplay between genetic predispositions (e.g., ApoE4 allele) and vitamin D levels may provide further insights into individualized prevention strategies (36, 37).

## 6 Conclusion

This comprehensive meta-analysis of 22 observational studies demonstrates a significant inverse association between serum vitamin D levels and dementia risk, with a linear dose–response relationship indicating a 1.2% reduction in risk for every 10 nmol/L increase in vitamin D. These findings support the potential role of maintaining adequate vitamin D levels in promoting cognitive health, particularly in populations with high prevalence of deficiency. However, causality cannot be established from observational data. Well-designed randomized controlled trials are needed to confirm these results, determine optimal vitamin D thresholds for cognitive protection, and clarify potential interactions with genetic and lifestyle factors. In the meantime, public health strategies to prevent vitamin D deficiency may represent a pragmatic approach to reducing dementia risk at the population level.

## Data Availability

The raw data supporting the conclusions of this article will be made available by the authors, without undue reservation.
