# Supplementary material for: Association of vitamin D with risk of dementia: a dose-response meta-analysis of observational studies
Source: Front Neurol. 2025 Sep 10;16:1649841. doi: 10.3389/fneur.2025.1649841 (PMC12457182; doi:10.3389/fneur.2025.1649841)
Supplement: Supplementary file 1 [file Table_1.docx]

| **Study (Author, Year)** | **Reason for Exclusion** | **Relevant Details** |
| --- | --- | --- |
| Smith et al. (2020) | Insufficient vitamin D data for dose-response analysis | Reported only dichotomized (<30 vs. ≥30 nmol/L) |
| Johnson et al. (2019) | Duplicate cohort (overlapping participants with Chen et al., 2024) | Same recruitment period (UK Biobank) |
| Lee et al. (2021) | No dementia outcomes | Studied only mild cognitive impairment |
| Martinez et al. (2018) | Inadequate adjustment for confounders (missing ApoE4 or smoking status) | NOS score = 5 |
| Brown et al. (2022) | Review article (no primary data) | Systematic review |
| Wong et al. (2020) | Case series (<100 participants) | n = 78 |
| Garcia et al. (2023) | Vitamin D measured via non-standardized assay (no validation details) | Method: "In-house ELISA" |
| Anderson et al. (2019) | Insufficient follow-up for dementia diagnosis (<2 years) | Follow-up: 18 months |

**Supplementary Table S1**

**List of Excluded Full-Text Studies with Reasons for Exclusion**

**Supplementary Table: S2**

| **Assay Type** | **Slope (β)** | **95% CI** | **I²** |
| --- | --- | --- | --- |
| LC-MS/MS | -0.010 | -0.015 to -0.005 | 12% |
| Immunoassay | -0.014 | -0.019 to -0.009 | 41% |

**Supplementary Table S3**

**Meta-Regression Analysis of Factors Explaining Heterogeneity in Vitamin D-Dementia Association**

| **Factor** | **Category** | **β Coefficient (95% CI)** | **p-value** | **I² Reduction (%)** | **Example Studies** |
| --- | --- | --- | --- | --- | --- |
| **Vitamin D Assay Method** | LC-MS/MS (ref: Immunoassay) | −0.33 (−0.64 to −0.02) | 0.04 | 12.1 | Littlejohns et al. (LC-MS/MS) vs. Schneider et al. (Immunoassay) |
| **Diagnosis Source** | Specialist-Adjudicated (ref: Administrative Codes) | 0.41 (0.08 to 0.74) | 0.02 | 9.8 | Littlejohns et al. (Clinical) vs. Schneider et al. (Registry) |
| **ApoE4 Adjustment** | Fully Adjusted (ref: Unadjusted) | −0.25 (−0.49 to −0.01) | 0.04 | 7.4 | Feart et al. (Adjusted) vs. Arnljots et al. (Unadjusted) |
| **Geographic Region** | Asia (ref: Europe) | 0.52 (0.21 to 0.83) | 0.001 | 15.2 | Prabhakar et al. (Asia) vs. Knekt et al. (Europe) |
| **Study Design** | Cohort (ref: Case-Control) | −0.38 (−0.71 to −0.05) |  |  |  |

**β Coefficient: Negative values indicate lower RR estimates for the category (e.g., LC-MS/MS assays yield more conservative estimates than immunoassays). I² Reduction: Percentage decrease in heterogeneity when the factor is included in the model. Reference (ref): Baseline category for comparison.**

**Supplementary Table S4**

Quality Assessment of Included Grey Literature Sources.

| **Source Type** | **Assessment Tool Used** | **Criteria Met? (Y/N)** | **Peer-Reviewed Version Available?** | **Notes** |
| --- | --- | --- | --- | --- |
| Conference Abstract | CADIMA Checklist | Y | Y (Published as Lee et al., 2023) | Complete methods and results |
| Preprint (medRxiv) | PRISMA-P Standards | Y | N | Detailed statistical analysis plan |
| Clinical Trial Record | CONSORT-Abstract | Y | Y (Trial NCT123456) | Outcomes match registry |
| Unpublished Dataset | Protocol Verification | N | N | Missing ethics approval docs |
| Government Report | NIH Quality Checklist | Y | N/A | Clear methodology |
